# Fully-automatic blood-typing chip exploiting bubbles for quick dilution and detection

**DOI:** 10.1063/5.0006264

**Published:** 2020-04-14

**Authors:** Ken Yamamoto, Ryosuke Sakurai, Masahiro Motosuke

**Affiliations:** 1 Department of Mechanical Engineering, Tokyo University of Science, 6-3-1 Niijuku, Katsushika-ku, Tokyo 125-8585, Japan; 2 Research Institute for Science & Technology, Tokyo University of Science, 6-3-1 Niijuku, Katsushika-ku, Tokyo 125-8585, Japan

## Abstract

A compact, fully-automatic blood-typing test device is developed. The device conducts sequential processes of whole-blood dilution, homogenization, and reaction with reagents. The lab-on-a-chip device can detect the weakest reaction between red blood cells (RBCs) and reagents even without using optics such as a camera and detector. This high sensitivity is achieved by implementing 50-*μ*m-thick reaction chambers in which a clear contrast between the RBC agglutinations and non-reacted RBCs can be obtained. The dilution and the homogenization are enhanced by injecting bubbles into the microchannel so that the test result can be obtained 5 min after the test start. With an assumption that the device will be used by medical staffs, the device is designed to require minimum operation for the users, namely, loading whole blood, starting pumps, and looking inside the reaction chambers by their eyes to observe the test result. As the device is applicable to the cross-matching test by mixing RBCs with serum instead of the reagents, it is expected that the device provides not only the quick blood-typing but also a safer and quicker blood transfusion in emergency rooms.

## I. INTRODUCTION

Blood conveys nutrients, oxygen, and metabolic wastes to and from cells and organs.^1^ Heavy bleeding is, therefore, a life-threatening event, and a prompt transfusion is required for this case to maintain vital activity. In early days of the blood transfusion history, severe adverse effects, which include fatal accidents, were frequently occurred. In the 17th century, it was found that there are dissolution and agglutination of red blood cells (RBCs) for some transfusion cases.^2^ The cause of these reactions was revealed in the 1900s by Landsteiner^3^ who found four types of blood, which contain different combinations of antigen and antibody. Owing to these findings, the blood-typing test was recognized to be a prerequisite to transfusion to prevent the adverse effects such as an acute kidney failure or an anaphylactic shock. In addition to the blood-typing test, the cross-matching (matching test of the patient and the transfusing blood) tests prior to the transfusion are required nowadays for the safe transfusion.

According to the types and amount of antigen and antibody, blood is classified into more than 30 different types.^4,5^ In general, blood types are categorized by the ABO (A, B, O, and AB) and Rh (Rh+ and Rh−) blood types.^6^ The ABO blood type is determined according to the antigen on the red blood cells (RBCs) and the antibody in the plasma, whereas the Rh blood type is determined whether the RBCs have the D antigen (Rh+) or not (Rh−). The blood-typing test, which is widely performed in medical centers and blood banks, exploits these features by observing the antigen–antibody reaction of sample RBCs with different antibody reagents, in which the RBCs agglutinate when specific combinations of the antigen and the antibody are mixed.^1,6,7^

Compared to its simple principle and frequency of performance, the conventional blood-typing tests cost relatively high because they require specialized techniques (e.g., centrifuge and precise pipetting) and take more than 30 min.^8^ While the working environment of medical staffs is of particular problem worldwide, these 30 min are the cost to be cut. Automation is considered to be one of the solutions to this situation. However, although some fully-automatic blood-typing systems were developed^9,10^ and they can reduce the human cost significantly, there still is a problem of the economic cost.

From a perspective of the cutting cost of the blood typing, lab-on-a-chip devices have advantages owing to their ability to reduce the reaction time and to integrate simplified and automated systems.^11,12^ Paper-based microfluidic devices are popular examples of the lab-on-a-chip blood-typing devices.^8,12–15^ Zhang *et al*.^8^ exploited the reaction of bromocresol green (BCG) with serum protein to exhibit teal blue in their device when the blood-type antigens exist, while the device indicates brown when there are no reactions. Li *et al*.^15^ developed a blood-typing device that indicates the test result as millimeter-scale characters patterned by controlling the wettability of the device surface. The device is designed to accumulate the RBCs on the characters to color them red only when they are agglutinated.

While the capillarity, which is the driving force of the paper-based microfluidics, is beneficial to the simple devices, some other external forces are required for more complicated analyses. Kim *et al*.^16^ integrated chaotic micromixers^17,18^ for mixing the blood and antibody reagent, reaction chambers, and microfilters that trap the agglutinations in a polydimethylsiloxane (PDMS) microdevice. The device automatically performs the mixing and reaction once the blood and antibody reagent are infused by pumps. Chen *et al*.^19^ developed a simple blood-typing device that does not require any pumps: in their system, a drop of sample blood is loaded together with phosphate-buffered saline (PBS) into a hole, and the (PBS-diluted) sample is introduced into the reaction chambers by a screw turned on the hole. Park and Park^20^ employed the finger pressure as the driving force. Because these devices are made of polymers, the device fabrication process is simple and reproducible. Moreover, these devices have also remarkable features that the results can be confirmed by naked eyes.

As many cost-efficient devices were developed, one of the hottest issues on this field is now shifting to the sensitivity: they cannot exclude the possibility of unrecognizing tiny (weak) agglutinations,^21^ which leads to false decisions. Because some subtypes of the ABO blood type^22^ show a very low agglutination level, increasing the sensitivity is essential to prevent the false decisions. To improve the sensitivity, methods such as the lab-on-a-disk system,^23–25^ the surface plasmon resonance (SPR),^26–31^ the image-analysis methods,^32–34^ and electrical impedance methods^21^ were developed. However, these methods have issues on simplification of fabrication and/or handling.

In this study, we develop a sensitive and cost-efficient reaction-detection method that is applicable to tests using red blood cells to free the medical staffs from manual handling and hour-long stand-by time. With the developed method, blood-typing tests are demonstrated in a device in which whole-blood dilution, reaction, and positive/negative indication are automatically performed. Exploiting peculiar behaviors of bubbles in a microscale, the method enables us to execute several sequential processes in the test automatically once the sample whole blood is loaded and the test is started.

## II. EXPERIMENTAL SETUP AND PROCEDURE

The concept of the device is achieving high sensitivity while user-friendliness and low cost are held. Four points stated below are paid particular attention:

(a) The system is fully automatic.
(b) Dilution of whole blood can be performed *inside* the device.
(c) The whole system is composed of a chip and pumps to keep it simple.
(d) The reaction result is observable by naked eyes while holding sufficient sensitivity.

We assume that the only processes that medical staffs have to do are loading fluids and push a “start” button. We also assume that there is enough amount (>10° ml) of blood when the fast blood typing is necessitated such as in emergency rooms (ERs).^35^

**FIG. 1.**
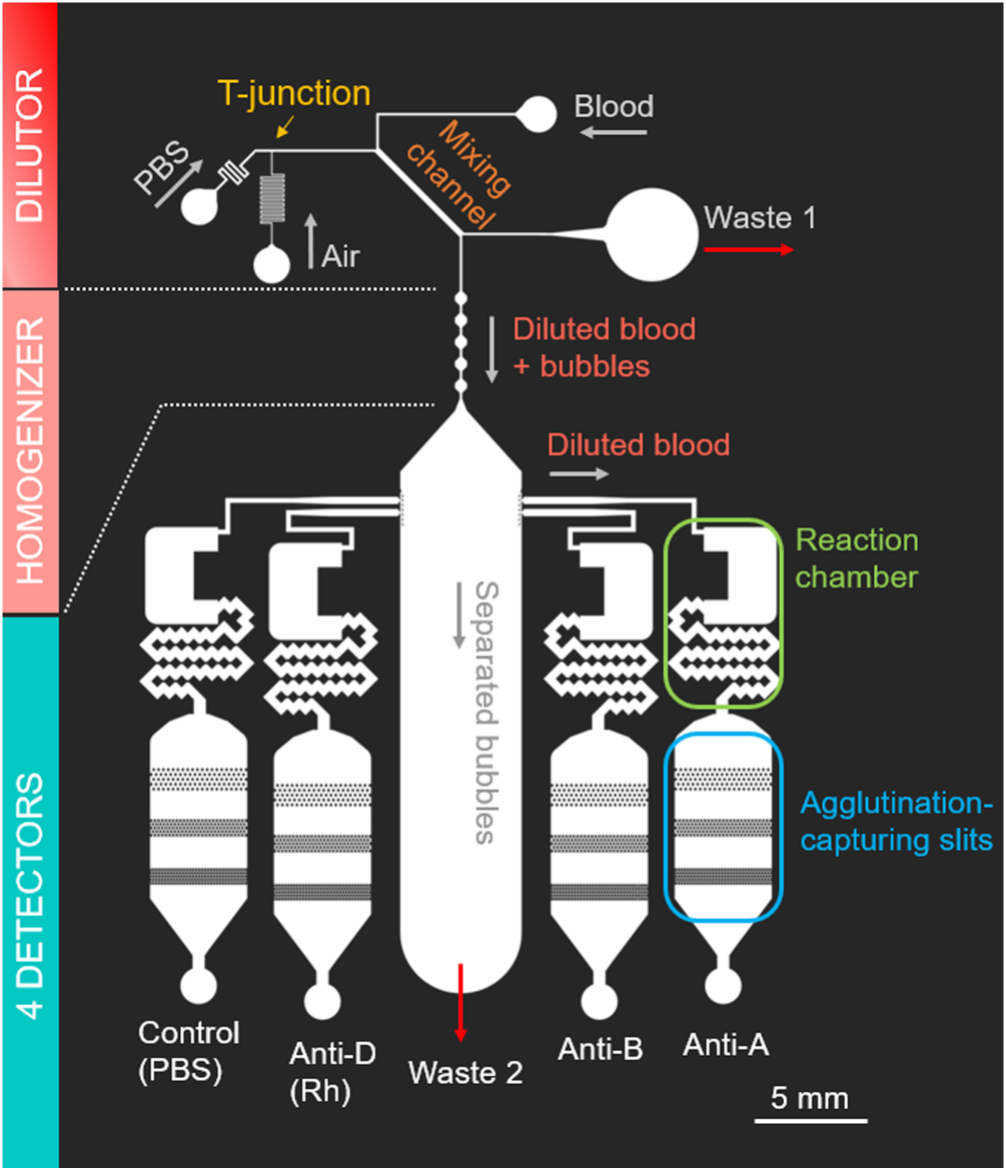
Whole design of the blood-typing chip. The device consists of a bubble generator (T-junction), a dilutor, a homogenizer, bubble separators, and detection chambers.

The whole design of the developed blood-typing device is shown in Fig. 1. The device size is approximately 3 × 5 cm^2^, and it consists of a dilutor, a homogenizer, and four detectors. The dilutor consists of three inlets (for whole blood, PBS, and air) and junctions. The device is made of polydimethylsiloxane (PDMS), which is plasma-bonded on a glass substrate. The PDMS channel is fabricated by the standard soft lithography method. The channel height was set to 50 *μ*m throughout the chip.

In the dilutor, the dilution (mixing) is performed in a few seconds by means of a microbubble motion. At the upstream region of a Y-junction where whole blood and PBS first contact, air bubbles are generated in the PBS flow at a T-junction (Fig. 1, top left) and transported to the Y-junction along with PBS. The mixture of PBS and the bubbles contacts with whole blood at the Y-junction and flow in the mixing channel (Fig. 1, top center) together. In the mixing channel, the bubbles intensively mix blood and PBS by generating disturbance of the mixing front.^36^ However, because of the low flow disturbance (in comparison with the convective mixing) in a microscale and an extremely low diffusion coefficient of RBCs, the concentration of the blood–PBS mixture has a spatial nonuniformity at the end of the mixing channel. Exploiting this feature, the device extracts half of the mixed solution whose concentration is lower than the average. At the downstream of the dilutor, the spatial uniformity of the solution is assured by the homogenizer in which intensive mixing occurs due to chaotic motions of the bubbles.^37^ The homogenizer consists of five in-line hexagonal chambers.

The channel widths of the dilutor components are 100 *μ*m for the PBS and blood channel, 50 *μ*m for the air channel, and 250 *μ*m for the mixing channel. Blood and PBS are supplied by two syringe pumps, whereas air is supplied by a pressure-based flow controller. Three flow conditions listed in Table I were tested to obtain different dilution ratios. Note that we chose air as the immiscible phase because oil showed an unfavorable interaction with RBCs. Moreover, we added the surfactant (Tween 20, 0.1 v/v%) to PBS (buffer phase) for better dispersibility. It was confirmed through the conventional manual test that addition of the bubbles and surfactant has no remarkable effect on the RBC agglutination.

**TABLE I.** Injection conditions of sample solutions.

Condition	Flow rate/pressure		
	PBS (*μ*l/h)	Air (kPa)	Blood (*μ*l/h)
1	400	16	400
2	600	18	350
3	750	20	500

**FIG. 2.**
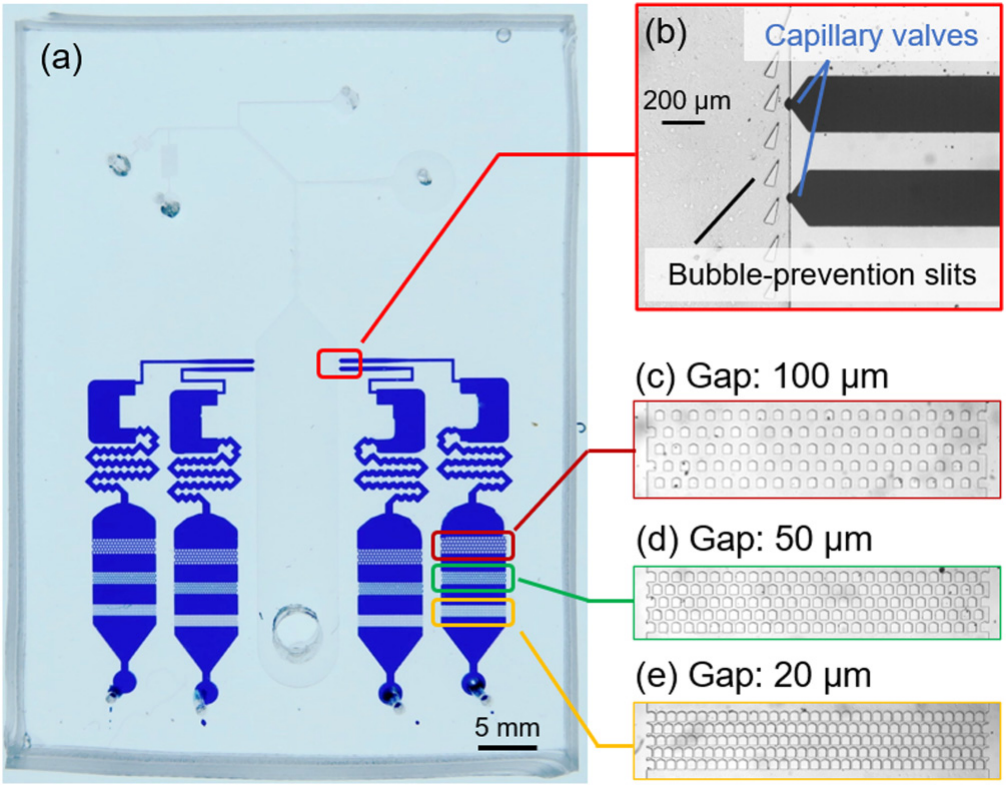
(a) Pre-loading of reagents in the reaction chambers (colored water is loaded for the demonstration). (b) Enlarged image of the capillary valves and bubble-prevention slits. The loaded liquid is held at the capillary valves. (c)–(e) Three slit lines for trapping the RBC agglutinations. Three lines have different gap widths of 100, 50, and 20 *μ*m (from upstream to downstream), respectively.

In the downstream region, the device has four detectors (Fig. 1, bottom). The detectors are designed to contain pre-loaded reagents and mix the diluted blood and the reagent as blood flows into the reaction chamber. The pre-loaded reagents are designed to fill the reaction chamber and standby at the chamber inlet by employing capillary valves [i.e., the chambers are filled by reagents, whereas the remaining part inside the device is filled with air, as demonstrated by loading colored water in Fig. 2(a)]. Inside the reaction chambers, the RBCs react with specific reagents and agglutinate as a result of the antibody–antigen reaction. The agglutinated RBCs are trapped at three slit lines where operators can recognize the result with naked eyes. In the present study, anti-A, anti-B, and anti-D reagents and PBS (as control) are pre-loaded into the respective detectors. Note that the diluted blood is separated from the bubbles prior to entering the detectors by triangle-shaped slits installed at the inlets of the detectors as shown in Fig. 2(b). The width of each detector is 5 mm. Each slit line has five staggered lines of pillars (100 *μ*m in width), and the gap spacing is 100 *μ*m (first line), 50 *μ*m (second line), and 20 *μ*m (third line), respectively [Figs. 2(c)–2(e)].

To investigate the sensitivity of the device, a reaction test with a diluted antibody reagent was also performed. In general, the agglutination level is categorized into five (4+, 3+, 2+, 1+, and 0) based on the strength of the agglutination (zero indicates “no agglutination”), and agglutinations of these levels can be reproduced by diluting the reagent with different dilution ratios.^21^ We employed reagents with dilution ratios of 1:1 (4+), 1:4 (3+), 1:8 (2+), 1:32 (1+), and pure PBS (0) to examine the sensitivity of the device (see Fig. S1 in the supplementary material).

## III. RESULTS AND DISCUSSION

### A. Evaluation of the dilution characteristics

**FIG. 3.**
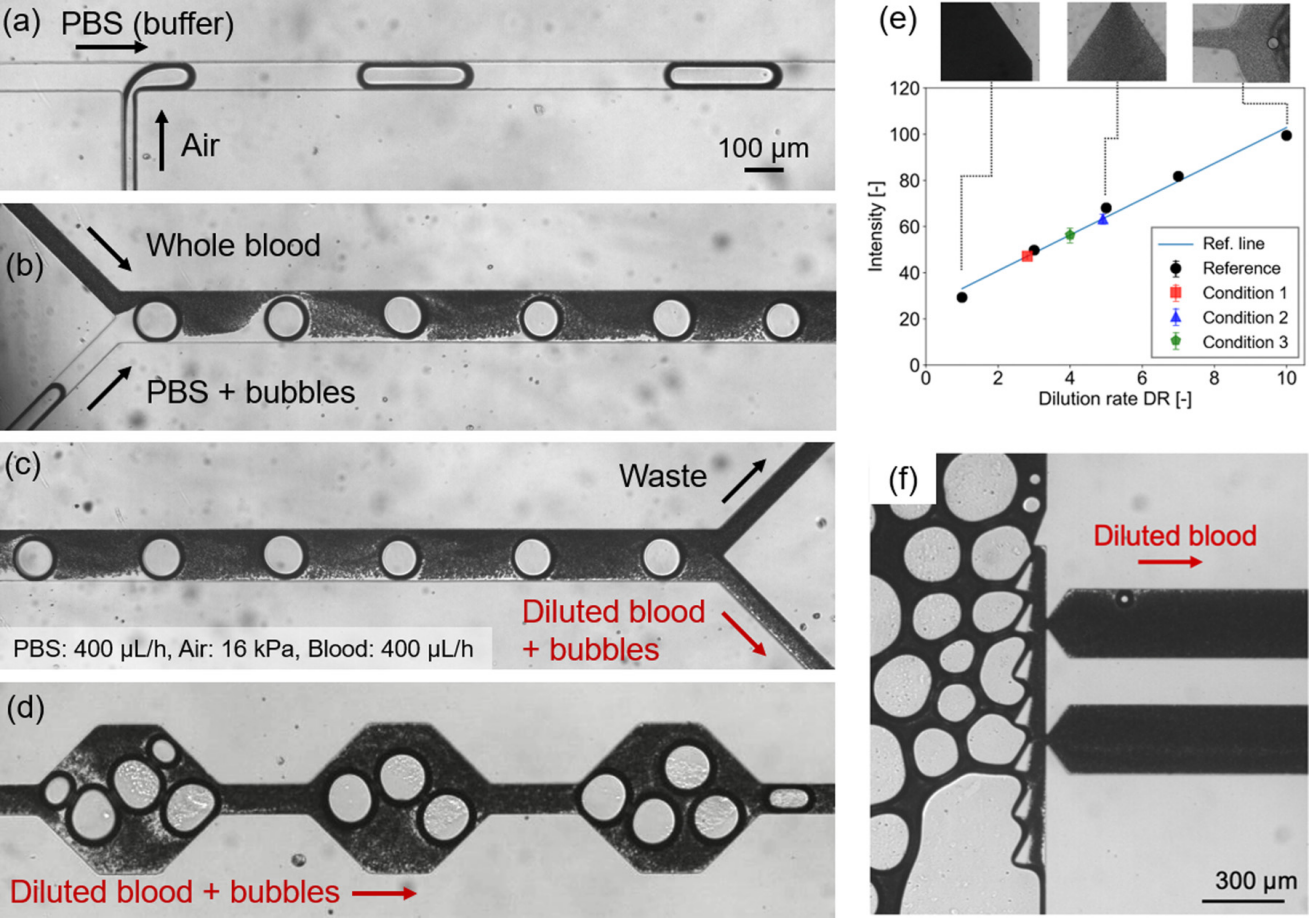
Microphotographs of (a) the T-junction for the bubble generation, (b) the inlet of the dilutor, (c) the outlet of the dilutor, and (d) the chaotic-mixing-type homogenizer. (e) Measurement of the dilution rate (DR) as a function of the image intensity in the device. Black plots represent the reference whose DR is pre-adjusted before loading to the device. (f) Microphotograph of the bubble separation by the bubble-prevention slits. Only the diluted blood is introduced into the reaction chambers.

The dilution mechanism using the immiscible phase^36^ is employed for the dilutor. A typical flow pattern formed by the introduction of the air phase is shown in Fig. 3(a). The introduced bubbles are carried to the Y-junction and work as the mixing enhancer by colliding with the mixing front in the mixing channel [Fig. 3(b)]. At the end of the mixing channel, half of the solution (whose concentration is higher than the other because there is a concentration gradient in the cross-sectional direction in the mixing channel) is discarded to speed up the test [Fig. 3(c)], and the rest of the diluted blood as well as the bubbles is transferred to the homogenizer to achieve a spatiotemporally uniform concentration with the assistance of the chaotic motion of the bubbles [Fig. 3(d)].

The concentration (dilution rate) of the blood can be adjusted by changing the flow conditions. Figure 3(e) shows the dilution rate (DR) at different flow conditions (listed in Table I) measured from the attenuation of the backlight illumination in the chip (the reference line was obtained with a preparatory-DR-adjusted solution). The result indicates that the dilution method can precisely adjust the DR in a range of 3×–5× for the examined conditions.

The diluted and homogenized blood is separated from bubbles and introduced into four detectors at the downstream region. The bubbles are successfully separated by the bubble-prevention slits at the junction of the main channel to the branches [Fig. 3(f)]. Blood inflows to the detectors are assured by the separated bubbles as they increase the hydrodynamic resistance in the center channel as the number of the bubble increases.

### B. Evaluation of the detector characteristics

**FIG. 4.**
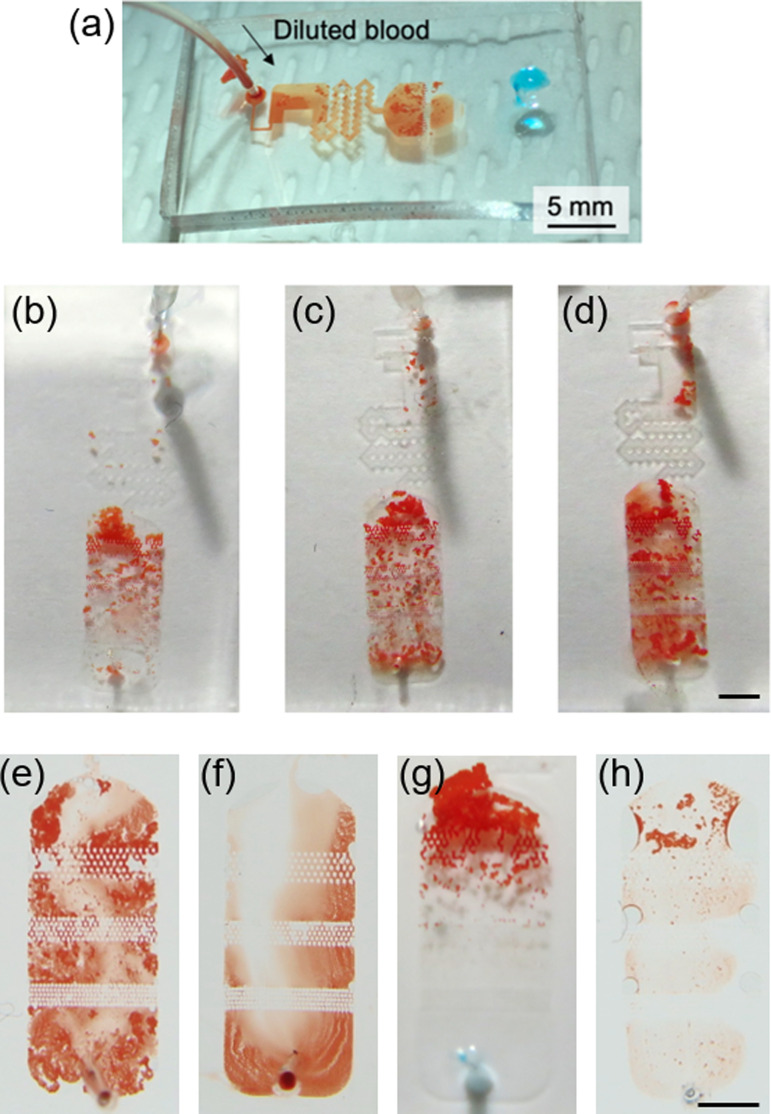
(a) Reaction test in a disconnected detector. Diluted blood is introduced by a syringe pump at 50 *μ*l/h into the detector where anti-A reagent is pre-loaded. (b)–(d) RBCs trapping result for the channel height of (b) 50 *μ*m, (c) 75 *μ*m, and (d) 100 *μ*m. The chambers are gently flushed by PBS for better visibility. (e)–(h) RBCs trapping result for the sample combination of (e) the whole blood and reagent, (f) the whole blood and PBS, (g) 5×-diluted blood and reagent, and (h) 10×-diluted blood and reagent. The chambers are gently flushed by PBS for better visibility. Non-indicated scale bars: 2 mm.

The detector consists of a reaction chamber and a detection chamber. We first evaluated the characteristics of the disconnected detector (Fig. 4) prior to its implementation in order to optimize the chamber geometry and the test conditions.

Figure 4(a) depicts the ongoing reaction in the disconnected detector. In Fig. 4(a), diluted blood is introduced from one inlet by the syringe pump into the detector, which is filled with the antibody reagent in advance. In this case, the flow rate was set to 50 *μ*l/h reckoning the flow rate at the dilutor and the flow distribution at the downstream region. Figure 4(a) clearly indicates that the reaction chamber increases the reaction surface by its channel-expanding shape, and the reaction is more enhanced by rotational flows at the corners of the chamber generated by its curved shape. These flows assist to provide a fresher reagent to the reaction front. By the supplied flow, the reacted RBCs are flown to the detection chamber and trapped at slits in the detection chamber.

The effect of the channel height on the detection was also evaluated. Figures 4(b)–4(d) show trapped RBCs in chambers having three different heights [(b) 50 *μ*m, (c) 75 *μ*m, and (d) 100 *μ*m]. The chambers were gently flushed by PBS for better visibility. Although the test condition except for the chamber height is the same and the chambers were flushed by PBS, contrast of the RBC agglutination and the background is weaker for the thicker chambers. This is presumably due to the thickness of the agglutination: because the RBCs mainly agglutinate laterally due to the confinement in the height direction, the agglutinated RBCs should have a high aspect ratio. Considering the characteristic size of the RBCs (∼10 *μ*m), typical thickness of the agglutination should be several tens of micrometers. Therefore, for lower chamber heights, the agglutinations could confine the chamber in the height direction, and its optical signal intensity becomes maximum. From these results, we chose the channel height of 50 *μ*m. Furthermore, the result also implies that the device does not require the flushing by PBS if the supplied blood is diluted sufficiently to distinguish the non-agglutinated and agglutinated RBCs. This topic will be further evaluated in Sec. III C. Note also that we set the smallest pillar spacing width to 20 *μ*m. This is because we observed clogging in some cases when the spacing is smaller than 20 *μ*m.

In addition to the above evaluation, the effect of the dilution ratio was also evaluated to optimize the visibility of the reaction result. Figures 4(e)–4(h) show reaction tests with differently diluted blood (whole blood, 5×, and 10× diluted blood). It indicates that the differentiation between the reacted [Fig. 4(e)] and non-reacted [Fig. 4(f)] cases is not clear if the whole blood is used. The differentiation becomes easier by diluting blood [Figs. 4(g) and 4(h)]. From these results, we set the DR to be 5× because it can provide more contact of the RBCs and the reagent while the clear contrast of the result is maintained, albeit 10× dilution is standard for the conventional blood test.

### C. Evaluation of the sensitivity

**FIG. 5.**
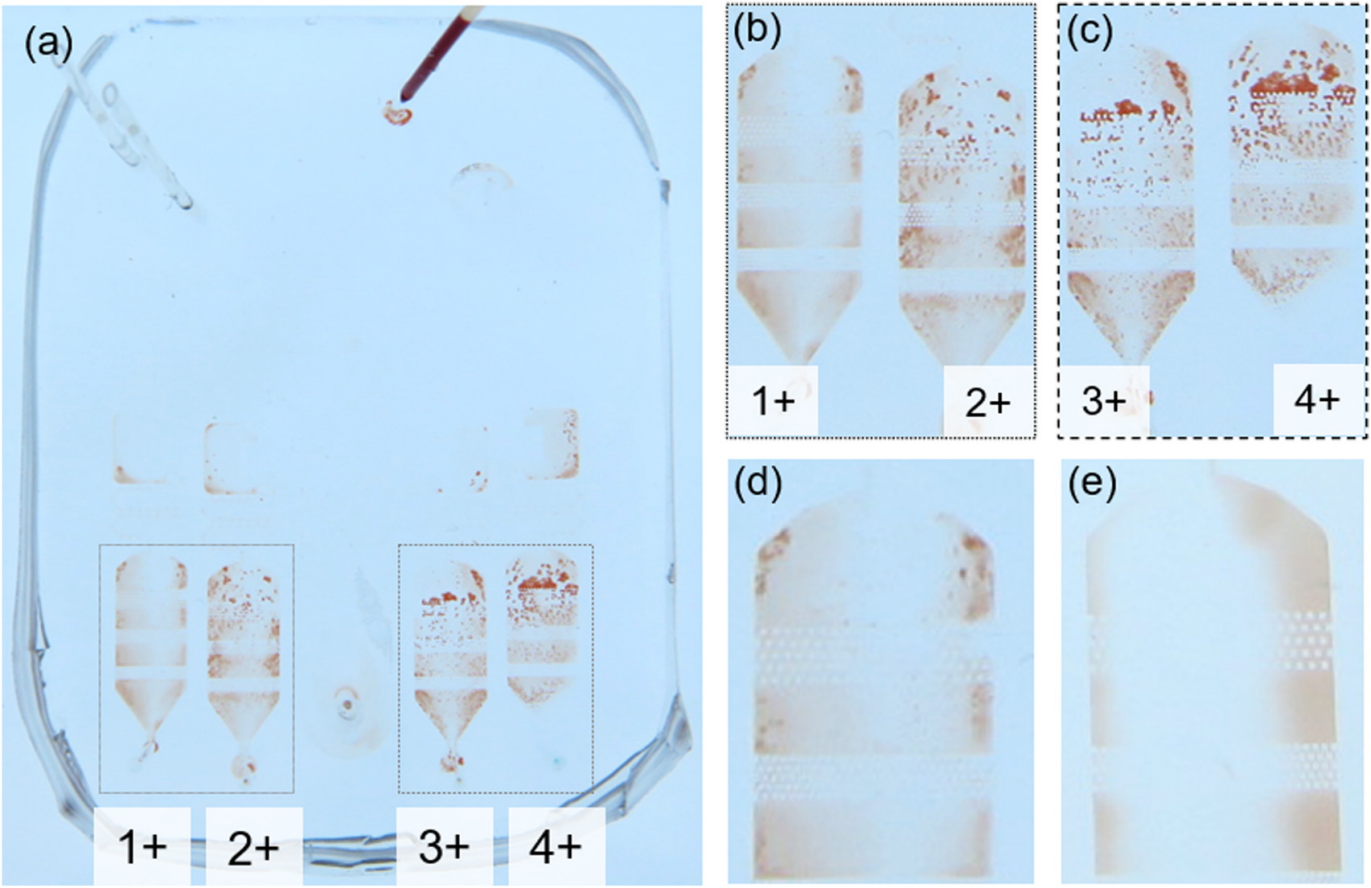
(a) Detection of different agglutination levels (A+). Respective agglutination levels are obtained by preloading PBS-diluted reagents (dilution rates of 1:32, 1:8, 1:4, and 1:1 for 1+, 2+, 3+, and 4+, respectively). (b) and (c) Enlarged views of the reaction chambers. Number of the RBC agglutination (red-colored area) indicates the agglutination level. (d) Enlarged image of the 1+ chamber. Small amounts of agglutinations are clearly observable for the agglutination level of 1+, whereas they are not observed for the (e) control (mixing of blood and PBS) experiment (image taken from another experiment).

After the considerations stated above, the dilutor and the detectors were implemented on a chip, and the sensitivity of the chip was evaluated by performing the reaction with different pre-loaded-reagent concentrations (see Fig. S1 in the supplementary material). Figure 5 shows a test result following the processes of the on-chip 5× dilution, homogenization, bubble separation, and flushing by PBS (blood and air supply were stopped, while PBS was kept supplying). The result indicates that the device can detect the 1+ agglutination and that the number of the RBC agglutinations are different for different agglutination levels: detectors for the higher agglutination levels contain a larger number of agglutinations. Moreover, the distribution of the agglutination in three slit lines implies that the size (width) of the agglutination does not depend strongly on the agglutination level (a typical width of several hundred micrometers). As mentioned in Sec. III B, the flushing by PBS is not necessarily for the test if a sufficient contrast between the agglutinated RBCs and the background blood is obtained. This condition holds for all the agglutination levels because the signal intensity from each agglutination is roughly the same and only the number of the signal determines the agglutination level. Therefore, the result indicates that the device has sufficient sensitivity without using any optical devices.

### D. Blood-typing test

**FIG. 6.**
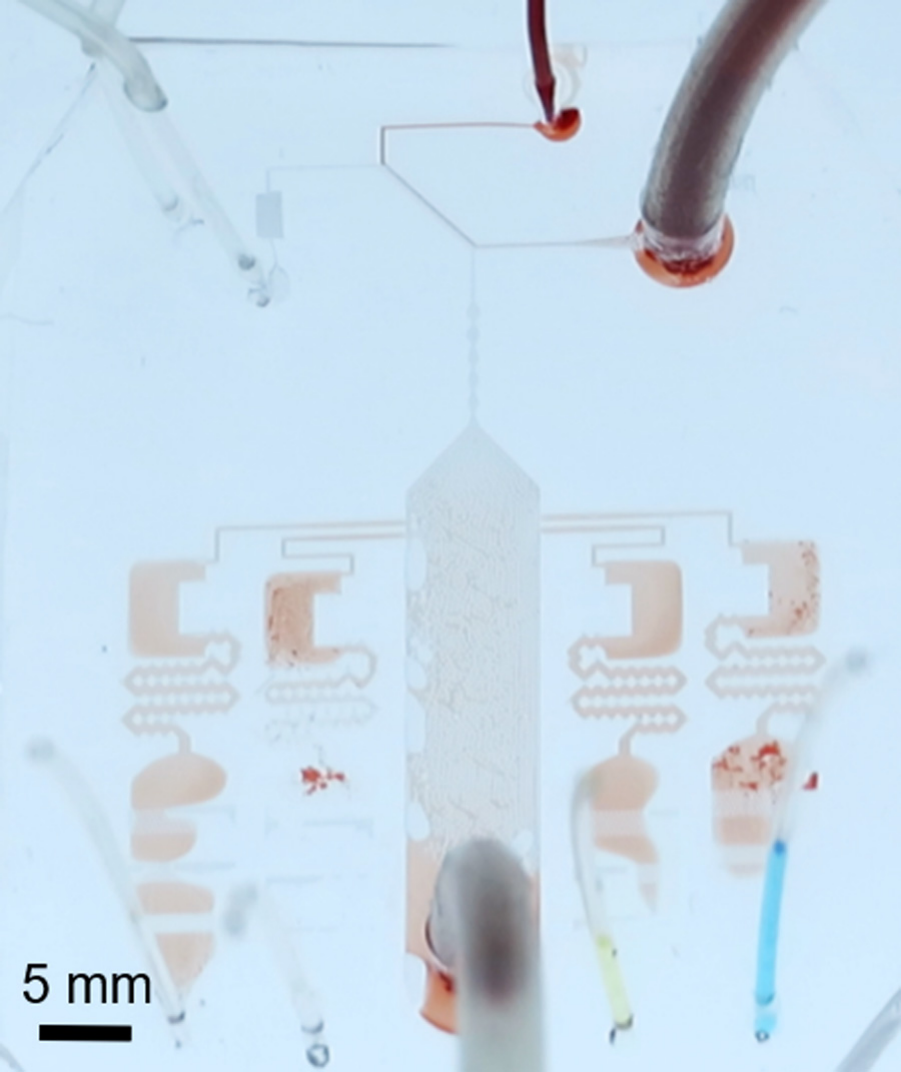
Blood-typing test (A+) at *t* ∼ 5 min. Detection chambers are for A, B, D, and control (PBS) from right to left. The reaction is positive in A and D, whereas it is negative in B (and control). Dilution, bubble separation, reaction, and the result indication are successfully performed.

The blood-typing tests were performed with the developed device and the optimized conditions. Starting from a condition that the reagents are pre-loaded and whole-blood, PBS-, and air-supply lines are connected to the device (*t* = 0), we observed that the diluted blood was introduced into the detectors in less than 1 min, and total time required to obtain one result was approximately 5 min. We performed ten blood-typing tests with blood collected from male donors (ages of 22–27). All experiments were performed in compliance with ethics and approved by the ethics committee of the Tokyo University of Science. The test returned ten correct results out of ten tests as one of them is shown in Fig. 6 (see also Figs. S2–S5 in the supplementary material).

## IV. CONCLUSIONS

A fully-automatic blood-typing test device was developed with a concept of user-friendliness. The device can return the test result within 5 min. For the device design, we weighed automation, simplicity, compactness, sensitivity, and an ability to obtain accurate results in a short time under an assumption that the device will be used by medical staffs in emergency rooms. For users who are not familiar with the lab equipment, the required operation was minimized to the sample loading and pump start. The minimization of the operation was achieved by implementing a dilutor, a homogenizer, and detectors on a chip. Moreover, exploiting bubbles also contributed to the size of the device (approximately 3 × 5 cm^2^) by reducing the attached equipment.

The bubble-motion-assisted dilution and homogenization system supply accurately diluted blood within a minute to the detectors. We examined several dilution conditions and found that five-time dilution is the best for the detection in the device, which is denser than the conventional manual test. This is because of the highly confined environment of the detector that enhances the contrast between the agglutinated and non-agglutinated RBCs. We also found that the size of the agglutination does not strongly depend on the agglutination level in the given condition, and only the number of the agglutination depends on the agglutination level. Because of this feature, the device exhibits high sensitivity that is sufficient to detect weak agglutinations (even level 1+) without any optics.

As Park and Park exploited common operation principles both for the blood typing^20^ and cross matching,^38^ our device is also applicable to the cross-matching test by mixing RBCs with serum instead of the reagents. With this feature, the device is expected to provide safer and quicker blood transfusion in ERs.

## SUPPLEMENTARY MATERIAL

See the supplementary material for more information on the agglutination level and the test result.

## AUTHOR’S CONTRIBUTIONS

K.Y. and R.S. contributed equally to this work.
